# Going deeper with health equity measurement: how much more can surveys reveal about inequalities in health intervention coverage and mortality in Zambia?

**DOI:** 10.1186/s12939-023-01901-x

**Published:** 2023-06-02

**Authors:** Andrea K. Blanchard, Choolwe Jacobs, Mwiche Musukuma, Ovost Chooye, Brivine Sikapande, Charles Michelo, Ties Boerma, Fernando C. Wehrmeister

**Affiliations:** 1 Institute for Global Public Health, University of Manitoba, R070-771 McDermot Ave, Winnipeg, R3E 0T6 Canada; 2 School of Public Health, University of Zambia, Lusaka, Zambia; 3 Monitoring and Evaluation Division, Ministry of Health, Lusaka, Zambia; 4 International Center for Equity in Health, Federal University of Pelotas, Pelotas, Brazil

**Keywords:** Health equity, Inequality measurement, Demographic Health Surveys, Child mortality, Intervention coverage, Reproductive, Maternal, Newborn and Child Health (RMNCH)

## Abstract

**Background:**

Although Zambia has achieved notable improvements in reproductive, maternal, newborn and child health (RMNCH), continued efforts to address gaps are essential to reach the Sustainable Development Goals by 2030. Research to better uncover who is being most left behind with poor health outcomes is crucial. This study aimed to understand how much more demographic health surveys can reveal about Zambia’s progress in reducing inequalities in under-five mortality rates and RMNCH intervention coverage.

**Methods:**

Using four nationally-representative Zambia Demographic Health Surveys (2001/2, 2007, 2013/14, 2018), we estimated under-five mortality rates (U5MR) and RMNCH composite coverage indices (CCI) comparing wealth quintiles, urban‐rural residence and provinces. We further used multi-tier measures including wealth deciles and double disaggregation between wealth and region (urban residence, then provinces). These were summarised using slope indices of inequality, weighted mean differences from overall mean, Theil and concentration indices.

**Results:**

Inequalities in RMNCH coverage and under-five mortality narrowed between wealth groups, residence and provinces over time, but in different ways. Comparing measures of inequalities over time, disaggregation with multiple socio-economic and geographic stratifiers was often valuable and provided additional insights compared to conventional measures. Wealth quintiles were sufficient in revealing mortality inequalities compared to deciles, but comparing CCI by deciles provided more nuance by showing that the poorest 10% were left behind by 2018. Examining wealth in only urban areas helped reveal closing gaps in under-five mortality and CCI between the poorest and richest quintiles. Though challenged by lower precision, wealth gaps appeared to close in every province for both mortality and CCI. Still, inequalities remained higher in provinces with worse outcomes.

**Conclusions:**

Multi-tier equity measures provided similarly plausible and precise estimates as conventional measures for most comparisons, except mortality among some wealth deciles, and wealth tertiles by province. This suggests that related research could readily use these multi-tier measures to gain deeper insights on inequality patterns for both health coverage and impact indicators, given sufficient samples. Future household survey analyses using fit-for-purpose equity measures are needed to uncover intersecting inequalities and target efforts towards effective coverage that will leave no woman or child behind in Zambia and beyond.

**Supplementary Information:**

The online version contains supplementary material available at 10.1186/s12939-023-01901-x.

## Background

Although Zambia has achieved notable reductions in maternal and child mortality rates, continued efforts to identify and target gaps are essential if Zambia is to reach national and global targets [1]. Improving access to quality health services is crucial for reducing mortality and morbidity for mothers and children [2, 3]. A key element of the Sustainable Development Goals’ (SDGs) third target is to effectively improve universal coverage of essential preventive interventions for Reproductive Maternal, Neonatal and Child Health (RMNCH). Relevant to leaving no one behind, health equity means understanding and addressing systematic or unjust inequalities in progress towards better health service access and outcomes between population groups based on their socio-economic position, residence, region and other stratifiers that characterise social hierarchy [4, 5].

The Zambia government’s vision since the 1990s has been to achieve, “equity of access to cost effective quality health care as close to the family as possible” [6]. Universal health coverage has since become a specific priority of the Ministry of Health’s National Health Strategic Plan since 2017. The availability of four national Demographic Health Surveys (DHS) since 2000 is an untapped resource for the systematic assessment of equity trends in RMNCH interventions and child survival in Zambia. If health policies and programmes are to truly leave no one behind, it is necessary to conduct more nuanced health equity measurement, particularly as inequalities appear to reduce, but may become concentrated among specific socio-economic groups and in particular places [7]. This can serve as a basis to further explore intersecting forms of social disadvantage, and their roots in broader socio-structural inequities and relations of power, that perpetuate worse health outcomes for these groups over time [8]. Monitoring distributional health inequalities is also essential in establishing whether policies and programmes equitably improved healthcare utilisation across the population [9].

This study aimed to understand how far we can take surveys in tracking progress in reducing inequalities in RMNCH coverage and mortality outcomes over the last two decades in Zambia. To do this, we conducted secondary analysis of the last four rounds of the cross-sectional Zambia Demographic Health Survey (ZDHS) to examine inequalities in mortality and coverage indicators using conventional equity measures including wealth, urban–rural residence and provinces. Then, we used multi-tier measures of disaggregation of mortality and coverage among and across wealth groups, residence and provinces over time, to further assess which groups have experienced greater improvements or are still left behind. In this paper, we present these inequality trends, and consider the relative plausibility, acceptability and added value of the conventional compared to newer multi-tier measures for revealing more about RMNCH inequalities in Zambia and beyond.

## Methods

### Data sources

The last four Zambia Demographic and Health Survey rounds in 2001/2, 2007, 2013/14, 2018 were used for this analysis. All surveys used multi-stage cluster sampling designs to obtain nationally representative data. Data was collected using standardised questionnaires among women of reproductive age living in the sampled households. The data collection methods for the ZDHS were described previously [10].

### Health indicators

The health impact indicator of under-five mortality rate (U5MR) was calculated using the syncmrates program in Stata. We obtained estimates of the number of deaths among children aged 0‐59 months out of 1000 live births, in the ten years preceding each round of the ZDHS to allow for larger sample sizes.

To capture intervention coverage across the continuum of care, we computed the Composite Coverage Index (CCI) [11], which combines key RMNCH indicators into a single measure. The CCI is a weighted average of coverage including eight essential interventions that represent broad categories or stages across the continuum of care (family planning, maternal and newborn care, child immunisation, and case management of childhood illness). Each continuum stage is given equal weight. The CCI is then calculated as:

$$CCI=\frac{1}{4} \left(FPC\mathrm{mo}+ \frac{ANC4+\mathrm{SBA}}{2}+ \frac{\mathrm{BCG}+2\times DPT3+MSL}{4}+CAREANYD\right)$$

where:

1. Reproductive care: Demand for family planning satisfied with modern methods among currently married women in need of contraception (FPCmo)
2. Maternal and newborn care: at least four antenatal care visits during last pregnancy (ANC4); and skilled birth attendance (SBA)
3. Childhood immunisation to children 12–23 months: Bacille Calmette-Guerin vaccination (BCG); three doses of Diphtheria-Pertussis-Tetanus vaccination (DPT3); Measles vaccination (MSL)
4. Management of childhood illness: Care-seeking for disease among children under five years with symptoms of fever, diarrhoea or suspected pneumonia in the last two weeks (CAREANYD)

This composite indicator is useful for comparative analyses within-country and over time [11, 12] and has also been shown to have a strong association with under-five mortality [13]. The standard errors for the CCI were computed using bootstrapping.

### Equity analyses

We estimated the under-five mortality rates and composite coverage indices, and associated 95% confidence intervals (CIs), in Stata version 16.0. First, we compared estimates using conventional inequality measures, including wealth quintile, place of residence (urban or rural) and province in ZDHS 2001/2, 2007, 2013/14 and 2018. Wealth scores were calculated by the team responsible for the ZDHS surveys using a principal components analysis of various assets such as household goods, dwelling materials, access to utilities, and land or livestock ownership [14]. These previously-derived household wealth scores were divided into quintiles within each survey, the first one representing the poorest 20% of households.

Secondly, we assessed inequalities in U5MR and CCI in more depth by dividing the ZDHS’ wealth scores into ten (decile) instead of five (quintile) groups. Then we used double disaggregation between socio-economic and geographic variables. This included deriving wealth quintiles based on the distribution of wealth scores in urban areas only, to compare the urban richest 20% to poorest 20%, and to those in rural areas. Then we created wealth tertiles using the ZDHS’ wealth score distribution for each province. Tertiles were used instead of quintiles to ensure more robust estimation at this level of disaggregation [15–17].

Equiplots and graphs were developed to visualise inequalities for mortality and intervention coverage by groups and over time [18]. To compare changes in equity over time, we computed absolute and relative summary measures for each stratifier, including rate differences and ratios for residence as a binary measure, weighted mean differences from overall mean (WMDM) and Theil indices for province as a nominal categorical variable, and slope indices of inequality (SII) and concentration indices (CIX) for wealth as an ordinal categorical variable [19]. WMDM shows the absolute difference of the health outcome estimate for each region (weighted by its sample population) from the national estimate. The Theil index is a relative measure that accounts for the proportion of the population in each region and the ratio of the estimate in each region to the national mean, applying a natural log function [19]. The SII is the absolute difference between the predicted outcome value of the individuals with the highest to lowest wealth, after regressing the mid-point of the cumulative proportion of the sample in each wealth category (using a score from 0 to 1 from most to least disadvantaged) against the health outcome estimate for that category. The CIX is calculated as twice the area between the curve and the line of equality on the plot of cumulative percentage of the sample ranked by socio-economic variable, starting with worst off on the x-axis and the cumulative percentage of the health outcome on the y-axis [13, 19, 20].

## Results

### Conventional inequality trends in U5MR and CCI by wealth quintile, urban–rural residence, and province

**Fig. 1:**
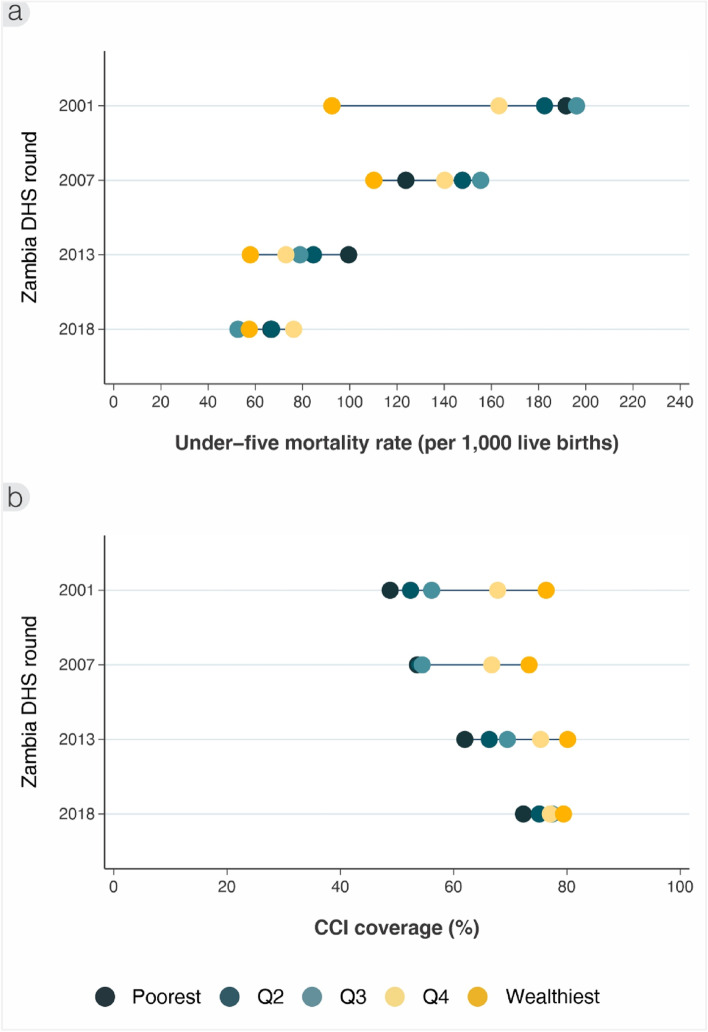
Inequality trends by wealth quintiles in (**a**) under-five mortality rate (per 1000 live births), and (**b**) composite coverage index (%), ZDHS 2001/2 to 2018

Overall, under-five mortality rates in Zambia reduced markedly, from 168 (95% CI: 161‐175) in 2001 to 64 (95% CI: 60‐69) per 1000 live births in 2018. Under-five mortality rate (U5MR) differences between wealth quintiles reduced markedly between 2001 and 2018 (Fig. 1a). In 2001, U5MR ranged from 192 per 1000 live births (95% CI: 177‐206) among the poorest quintile to 92 (95% CI: 78‐107) among the richest. By 2018, the U5MR was 67 per 1000 live births (95% CI: 59‐74) among the poorest quintile and was 57 (95% CI: 45‐70) for the richest. The large wealth gaps in 2001 are reflected in the SII of -108.9 per 1000 live births, and concentration index of -0.11. By 2018, the SII reduced to -4.5 per 1000 live births and the CIX was only -0.01.

Looking at the RMNCH composite coverage index, Zambia achieved large increases in CCI overall from 60% (95% CI: 59‐61) to 76% (95% CI: 75‐77) between 2001 and 2018 respectively. Inequalities in CCI by wealth also reduced greatly (Fig. 1b). In 2001 there was a large wealth gradient, from 49% (95% CI: 47‐50) among the poorest to 76% (95% CI: 74‐79) among the richest quintile. By 2018, the CCI was 72% (95% CI: 71‐74) among the poorest and 79% (95% CI: 77‐83) among the richest. The pro-equitable improvements are reflected in decreases in the SII from 34.6 to 8.1 percentage points between 2001 and 2018 respectively. Also, the CIX contracted from 0.09 to 0.02 respectively.

**Fig. 2:**
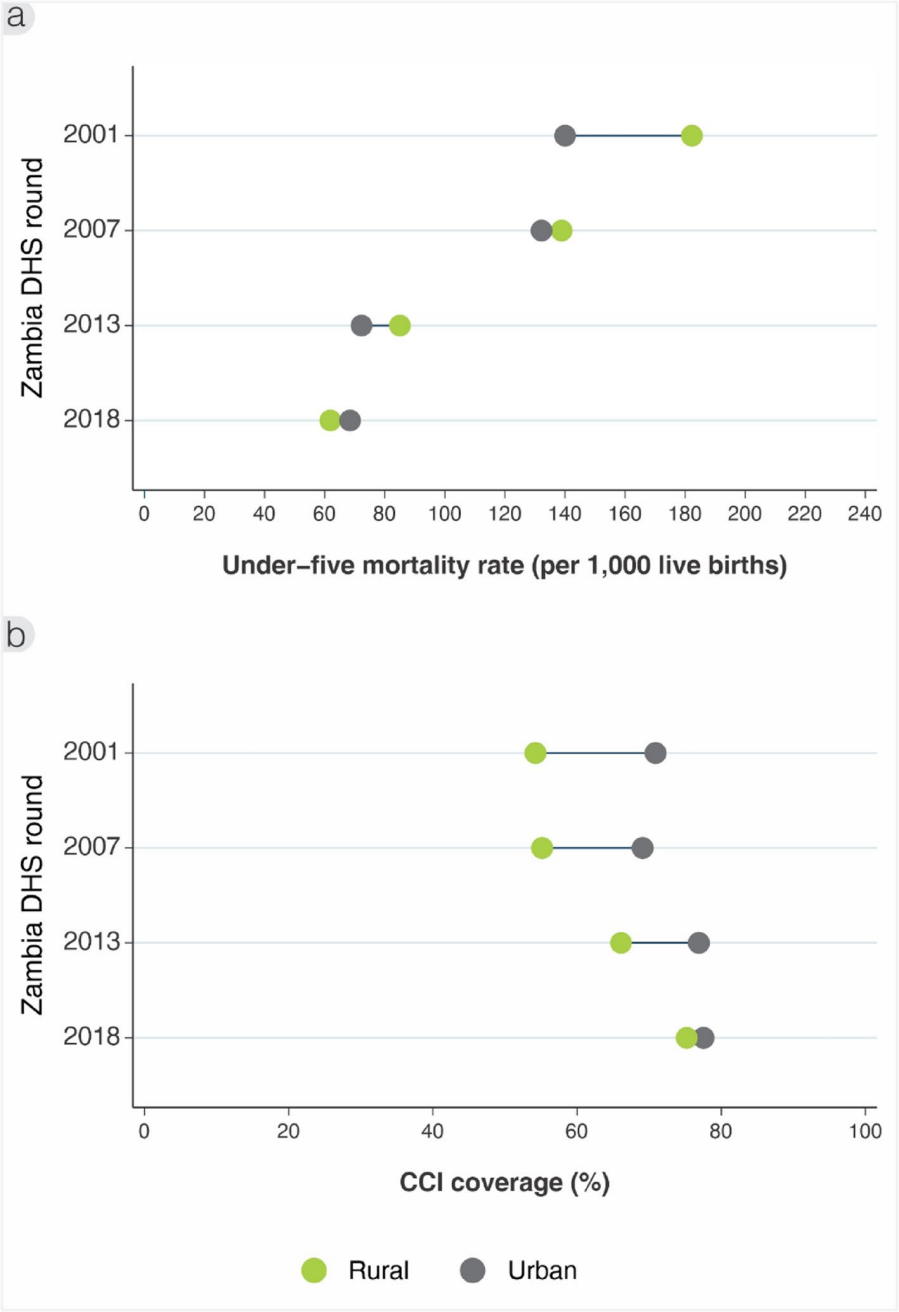
Inequality trends by rural–urban residence in (**a**) under-five mortality rate (per 1000 live births), and (**b**) composite coverage index (%), ZDHS 2001/2 to 2018

Comparing by residence (Fig. 2a), U5MR was estimated at 182 (95% CI: 174‐191) in rural areas compared to 140 (95% CI: 128‐152) in urban areas in 2001. By 2018, U5MR was 62 per 1000 live births (95% CI: 57‐66) in rural and 68 (95% CI: 58‐79) in urban areas. The absolute rate difference reduced from -42 to 6 per 1000 live births from 2001 to 2018 respectively, while the rural to urban ratio went from 1.3 to 0.9.

For CCI by place of residence (Fig. 2b), coverage started much lower among rural areas at 54% (95% CI: 53‐55) compared to urban at 71% (95% CI: 69‐73) in 2001. Yet by 2018, they were nearly equal at 75% (95% CI: 74‐76) and 78% (95% CI: 76‐79) respectively. This is reflected in the declining rate difference from 17 to 3 percentage points, and rate ratio from 1.3 to 1.0, between 2001 and 2018 respectively.

We also compared mortality trends in the ten provinces in Zambia (Supplementary Table 1). Absolute U5MR levels reduced in all provinces, and fastest among North Western, Central, Western and Northern provinces with an average annual rate of change above 6% each. The absolute inter-province inequalities reduced overall between 2001 to 2018 as well. This is reflected in the WMDM declining from 30 to 13 per 1000 live births. However, relative inequalities did not improve between 2001 and 2018 ZDHS, as reflected in Theil indices of 23 and 27 respectively. This is because some provinces were still lagging in 2018, including Luapula with U5MR at 110, Muchinga at 74, and Southern at 70.

Across provinces, the CCI inequalities improved greatly (Supplementary Table 2). Improvements were fastest among provinces with lower baselines, including Luapula, North Western, and Western provinces. This was reflected in a decreasing WMDM from 6.2 to 2.5 percentage points in absolute terms, while the relative Theil index reduced from 5.6 to nearly zero from 2001 to 2018.

### Multi-tier inequality trends in U5MR and CCI using wealth deciles, wealth quintiles in urban areas, and wealth tertiles by province

Given that inequalities in mortality and CCI in Zambia notably reduced according to conventional equity measures, how much more can the survey data help to identify those who continue to have the worst outcomes? We explored this using additional multi-tier measures of disaggregation, including wealth deciles to isolate the situation of the poorest 10%, and wealth groups specifically within urban areas or for each province.

**Fig. 3:**
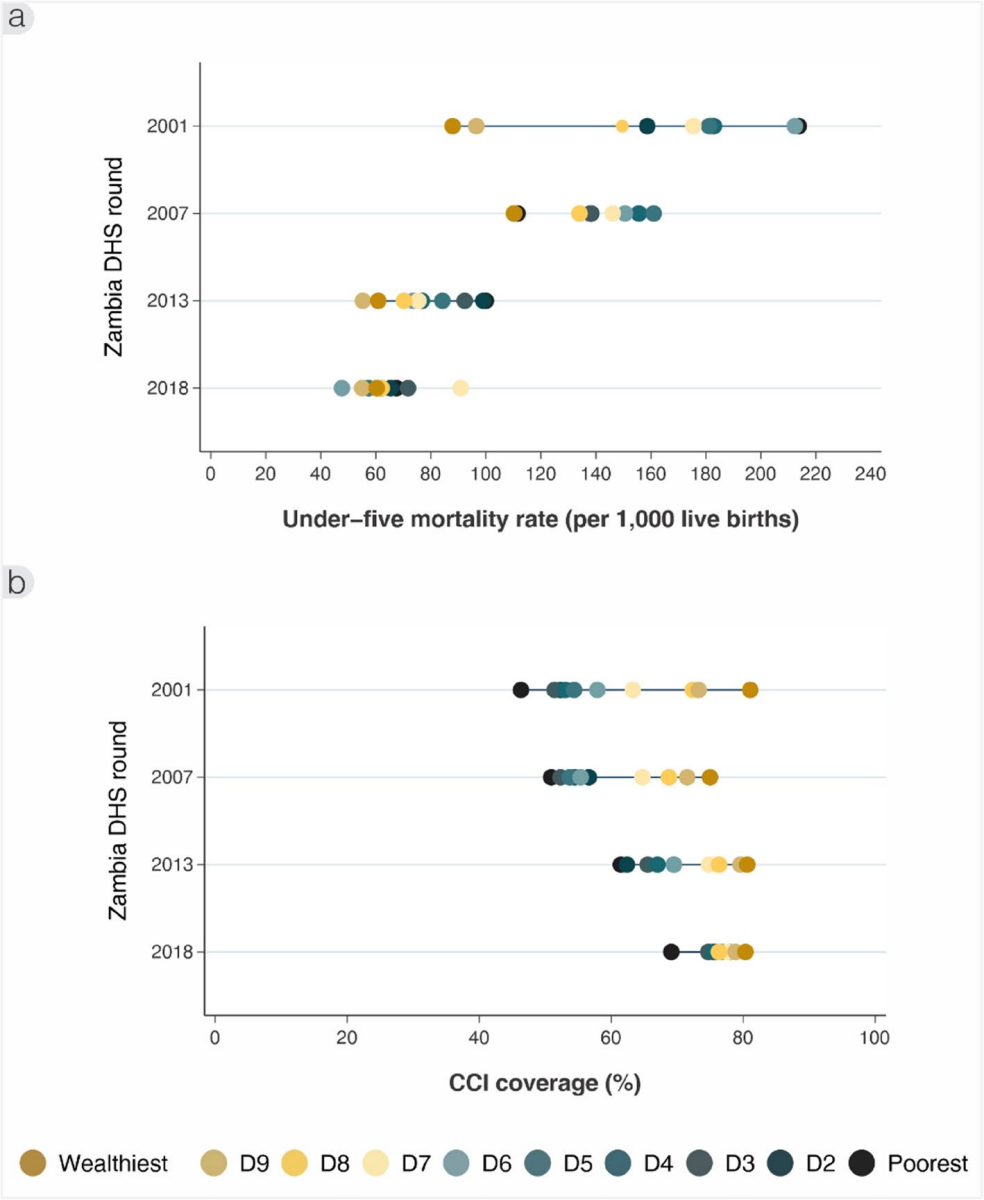
Inequality trends by wealth deciles in (**a**) under-five mortality rate (per 1000 live births), and (**b**) composite coverage index (%), ZDHS 2001/2 to 2018

Using wealth deciles (Fig. 3a), there were visible differences in U5MR in 2001 between the poorest (D1) and richest (D10) deciles (213 [95% CI: 191‐237] versus 88 [95% CI 59‐116] per 1000 live births, respectively). By 2018, the gradient appeared to reduce greatly, with an U5MR of 68 (95% CI: 56‐79) for the poorest 10% versus 60 (95% CI: 41‐79) per 1000 live births for the richest 10%. Gradients were somewhat uneven due to variable sample sizes. Nonetheless, sequential reductions from 2001 to 2018 were apparent using the complex summary measures that consider the proportion of the population in each of the ten groups: the SII declined greatly from -104.4 to -6.6 per 1000 live births, and the CIX from -0.11 to -0.02, respectively.

Looking at the CCI by wealth decile (Fig. 3b), a clear gradient was again evident in 2001 between the poorest (46%, 95% CI: 44‐48) versus richest deciles (81%, 95% CI: 78‐84%). By 2018, this became more equitable between the wealth deciles, though the poorest group still had lower rates (69%, 95% CI: 67‐71) than all other groups and especially the richest (80%, 95% CI: 77‐84). Pro-equitable improvements were evident in a declining SII from 35.6 to 8.3 percentage points, and CIX from 0.10 to 0.02 in 2001 and 2018 respectively.

**Fig. 4:**
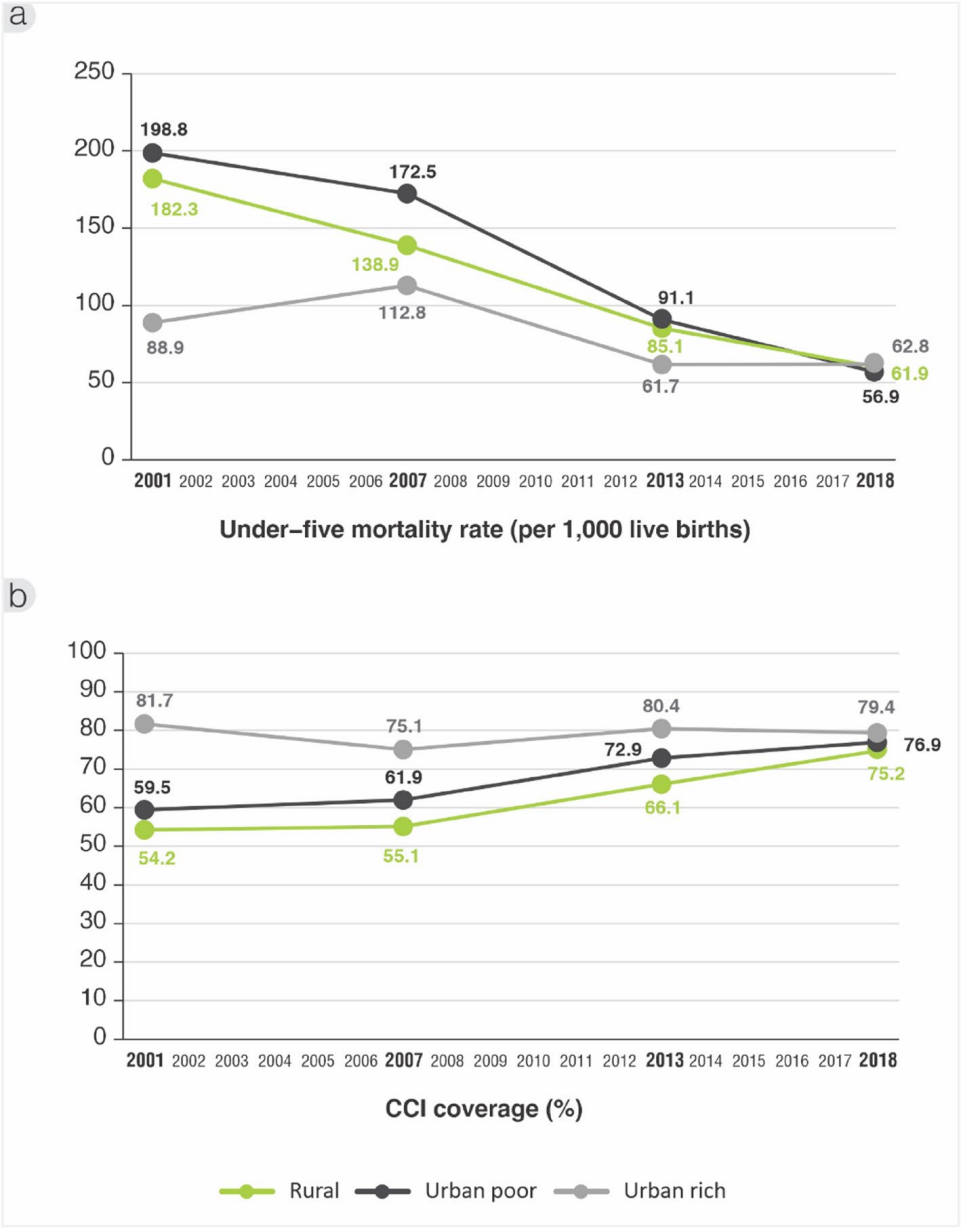
Inequality trends comparing urban poorest and richest wealth quintiles, and rural overall in (**a**) under-five mortality rate (per 1000 live births), and (**b**) composite coverage index (%), ZDHS 2001/2 to 2018

Given that the U5MR and CCI among rural areas caught up to urban areas in the conventional analysis, we also compared wealth groups within urban areas to explore whether the urban poor were being left behind richer groups (Fig. 4). The U5MR in 2001 was much higher among the urban poorest (198, 95% CI: 170‐228) than urban richest (89, 95% CI: 66‐112). By 2018, the U5MR for the urban poorest converged to nearly the same level as the urban richest, as well as the rural areas (Fig. 4a). In this way, the average annual rate of change over the 17-year period between surveys was -7% for the poorest, which was much higher than for the richest in urban areas at -2%, and even in rural areas overall at -6% (the rate of improvement was nearly equal across rural wealth groups).

**Fig. 5:**
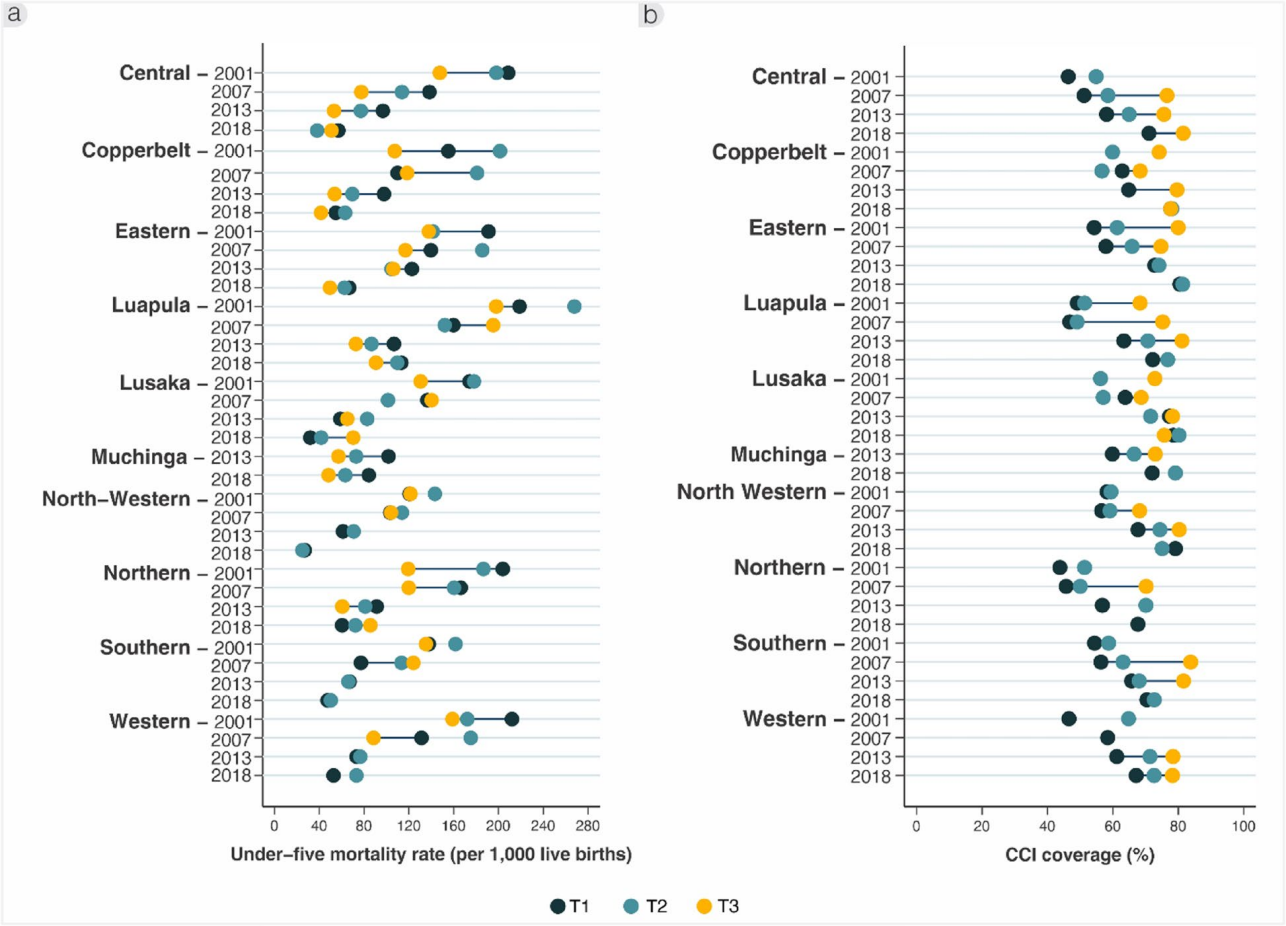
Inequality trends by province and wealth tertiles in (**a**) under-five mortality rate (per 1000 live births), and (**b**) composite coverage index (%), ZDHS 2001/2 to 2018

Turning to the CCI (Fig. 4b), we also see that coverage in 2001 among the urban richest (82%, 95% CI: 78–85) was nearly 30 points ahead of the urban poorest (59%, 95% CI: 56‐63). This in turn was slightly ahead of rural areas (54%, 95% CI: 53–55). By 2018, there was virtually no difference among the urban richest (79%, 95% CI: 76–83), urban poorest (77%, 95% CI: 74‐80), or rural areas (75%; 95% CI: 74‐76). This is reflected in the average rate of change in those 17 years of 1.5% for the urban poorest compared to nil for the urban richest, and 2% for the rural areas.

Finally, we compared U5MR by wealth tertile among each province (Fig. 5a, Supplementary Table 3). Inequalities in all provinces were wide in 2001, particularly in Central, Eastern, Northern and Western, where the poorest appeared to have the highest U5MR. However, U5MR seemed to reduce across wealth groups in all provinces by 2018, including for the poorest in those provinces with larger gaps to start. Summary equity measures showed less consistent patterns, and relatedly large confidence intervals.

CCI in each province was also compared by wealth tertile (Fig. 5b, Supplementary Table 4). Some estimates were not available when less common indicators within CCI (like care-seeking for children who are ill) had some zero values in smaller wealth and/or province groups. With available data, inequalities appeared large in 2001, particularly in Eastern, Luapula, and Western. By 2018, coverage improved for all and especially the poorer 60%, even in provinces with lower levels like Luapula and Western. To summarise, the SII reduced from 39 to 2 percentage points in Eastern, from 39 to 9 points in Luapula, and 26 to 17 points in Western between 2001 and 2018 respectively. The CIX also reduced between those years, from 0.09 to 0.003 in Eastern, 0.08 to 0.02 in Luapula, and 0.08 to 0.03 in Western respectively.

### Comparative assessment of equity measures

To assess whether multi-tier equity measures bring new insights compared to conventional stratifiers, we developed three assessment criteria based on whether their results were: 1) plausible, 2) reliable or acceptable, and 3) adding value for identifying those left behind, as follows:

1. *Plausible patterns:* consistent patterns by stratifier and over time in relation to what is logical or previously known
2. *Reliable based on confidence interval acceptability:* confidence intervals are not too wide, using the percent confidence interval divided by the related mean estimate. We used cut-offs of below 20%, 20‐40%, and over 40% to approximate good, fair and poor precision respectively (Supplementary Table 5)
3. *Providing new insights on who is left behind:* added value for capturing the situation of the most disadvantaged compared to the least disadvantaged population sub-groups (e.g. bottom 10% or 20% vs. richest 10% or 20%), given the spread across groups and/or their distance from the national average

Using these criteria (as summarised in Table 1), mortality estimates using wealth deciles were plausible but with less certainty or added-value compared to quintiles. As the number of deaths reduced over time, the distance of the confidence intervals to the mean was relatively greater across deciles in 2018 than 2001 (and for some groups like decile 7), leading to some instability in the gradients. Both deciles and quintiles illustrated that as U5MR started higher overall in 2001, there was a top-pattern of inequality where the richest 20% were much better off than all other groups. However, as national mortality levels fell, the gap between the second richest 20% (Q4) and richest 20% (Q5) reduced noticeably from 70 to 19 units. By 2018, the poorest quintile (or bottom two deciles, not only the bottom decile) caught up to others as inequalities noticeably reduced. Differences between wealth quintiles within urban areas also revealed new insights, which were plausible (clear gradients that reduced) and with acceptable uncertainty for nearly all comparisons (except the urban-richest in 2018, a smaller group among whom mortality was lowest). More than the other measures, wealth tertiles within provinces provided less insights due to high uncertainty in the estimates, making it harder to discern distinct wealth inequality patterns over time in most provinces.

**Table 1:** Comparing equity measures using assessment criteria for mortality and coverage outcomes

	**Under-five mortality rate**			**Composite coverage index**		
	**Plausible**	**Acceptable uncertainty**	**Added insights**	**Plausible**	**Acceptable uncertainty**	**Added insights**
**Conventional measures**						
*Residence*	Yes	Yes	Yes	Yes	Yes	Yes
*Province*	Yes	Yes, except smallest samples	Yes	Yes	Yes	Yes
*Wealth quintile*	Yes	Yes	Yes	Yes	Yes	Yes
**Multi-tier measures**						
*Wealth decile*	Yes	No, for some groups	Yes	Yes	Yes	Yes
*Residence by wealth quintile*	Yes	Yes	Yes	Yes	Yes	Yes
*Province by wealth tertile*	No, for smaller samples	No	No, for smaller samples in 2018	Yes	Yes	Yes, except some provinces missing data

CCI estimates using wealth deciles appeared to be plausible, acceptable and providing added-value compared to quintiles. The large CCI inequalities in 2001 reduced by 2018 as gaps between the richer two wealth deciles (D9 and D10) dropped from 7.8 to 1.5 percentage points, and similarly between the wealthiest two quintiles (Q4 and Q5) from 8.6 to 2.4 points. Conversely, the difference between the poorest two deciles remained, causing a bottom inequality pattern to emerge as national levels dropped. The gap between the poorest two quintiles closed in this time, suggesting deciles added new insights. CCI estimates for urban residence by wealth also satisfied all our criteria. CCI estimates compared in each province by wealth tertile were plausible and provided new insights in most cases. However, CCI could not be calculated for the richest tertile in provinces with relatively poorer populations (and for the poorer in the relatively richer provinces), due to insufficient samples for some indicators within the CCI like care-seeking for childhood illnesses. Summary measures could be calculated even with two tertiles, but confidence intervals could not be produced, presenting a different challenge for CCI as a composite indicator than for U5MR.

## Discussion

This study examined trends in U5MR and CCI inequalities in Zambia using conventional and multi-tier equity measures between women in different wealth groups, residing in urban or rural areas and provinces between 2001 to 2018. We sought to understand how much more demographic health survey data can tell us about who has experienced improvements or been left behind in RMNCH intervention coverage and mortality in Zambia. Our in-depth analyses found that newer approaches to disaggregation can often tell us more about who is being left behind, including some provinces, the poorest one or two wealth deciles, and the urban poor. However, this depended on the measures' acceptability and sometimes plausibility, which were affected by sample size and availability of data in different sub-population groups. This was especially true with under-five mortality rates, an increasingly less common event over time, and sometimes for composite coverage index when some of its components did not have enough cases with multiple disaggregation. We now discuss our findings in light of related research measuring health inequalities in RMNCH, and consider the implications for better identifying groups who are most left behind in Zambia and beyond.

Our study assessed three criteria to compare inequality measures’ utility for revealing which population sub-groups have higher mortality rates or lower intervention coverage, including which are most plausible, acceptable, and adding new insights. First, wealth deciles provided plausible, new insights for both U5MR and CCI compared to wealth quintiles, but were less precise for U5MR than CCI especially in later rounds when rates reduced. Others have previously assessed if wealth deciles tell us more than quintiles about inequalities by comparing changes in health outcomes between the top two and bottom two wealth groups [21]. As U5MR reduced and inequalities dropped by 2018 in this study, it was the bottom 20% and not 10% who were left behind, suggesting quintiles were sufficient. Wong et al.’s analysis of child stunting found that the poorest 10% were left behind in countries where levels reduced [21]. U5MR differences between the bottom two wealth deciles here may be less perceivable with smaller samples than for stunting. In contrast, wealth inequalities in CCI went from a top-pattern and large degree of inequality in 2001 when Zambia started at lower national levels [21, 22], to a bottom pattern of inequality where the poorest 10% were most left behind by 2018 [23]. This was similar to Wong et al.’s example of skilled birth attendance, one of the interventions within the CCI [21]. Thus, deciles provided further insights than quintiles for CCI. At the same time, richer groups never surpassed a CCI of 80%, suggesting that efforts to expand coverage are needed across wealth groups to maintain Zambia’s equity gains. The complex summary measures (SII and CIX) that considered all wealth groups’ populations did not differ between quintiles or deciles at any time point for U5MR or CCI, as others have found [21].

Double disaggregation further allowed us to examine how the poorest in urban areas or different provinces fared over time. Under-five mortality and CCI levels among the urban poorest improved faster than the richest, almost as much as for those living in rural areas, which differs from earlier analyses where the rural poor consistently lagged behind urban residents in Zambia and elsewhere [24–26]. Our related study to understand the greater reductions in U5MR for rural and poorer residents in Zambia pointed to a range of influencing factors, including the broad policy reforms that Zambia enacted to expand services for malaria and HIV/AIDS prevention, and RMNCH, with related increases in availability of rural health posts and personnel at facilities, as well as the efforts of community health volunteers to bring these services as close to families as possible [27]. Yet further attention is needed to understand who is not being well-served in spaces like urban unplanned settlements where socially disadvantaged groups often experience a combination of adverse living conditions and limited access to good quality health or other services [28–30]. As urban rich groups started with fairly high levels of CCI in 2001 ZDHS, continued improvements may have been harder to achieve, while their U5MR had more room to fall in this period. This may also be related to our previous findings that Zambia’s health policies and programmes focused greatly on expanding access to RMNCH services for rural and poorer populations, compared to urban and rich groups [27].

Our analyses were also able to show that wealth gaps in U5MR closed in every province over time, but inequalities seemed to remain higher in areas with higher U5MR levels like Luapula and Muchinga, though insights were limited by the imprecise estimates. For CCI, it appeared that inequalities reduced as coverage improved faster among poorer than richer groups (although some of the provinces did not have estimates for all groups). When using non-ordinal stratifiers like province, the lower precision of estimates for some provinces was less inhibitive for gaining insights from the others, compared to ordinal stratifiers like wealth that require comparisons across all groups. Still, summary measures like weighted differences from the mean difference or Theil indices to track the extent of subnational inequalities were less meaningful for those with smaller sub-groups.

We now consider this study's findings in relation to previous research tracking equity trends in Zambia and the Sub-Saharan Africa region. The inequality trends using different measures in this study showed that as Zambia’s mortality levels reduced between ZDHS 2001 and 2018, inequalities narrowed both absolutely and relatively. Another analysis of U5MR by wealth quintile comparing the ZDHS 2001 and 2013 DHS rounds (5 years preceding) showed that absolute but not relative inequalities reduced [31]. This is common when mortality levels drop but not drastically [32, 33]. Finding lower relative inequalities from ZDHS 2013 to 2018 (midpoints 2008 and 2013) indicates that U5MR dropped more quickly among the poorest relative to the richest (and absolutely) in the latest survey period compared to previous. Others found that Zambia had lower inequalities than all but five out of the 20 countries included from its region, as Zambia’s poorest (but not richest) had lower rates than the regional average in the most recent period [31]. Maintaining the same or faster average annual rates of change among poorer and rural groups (-6.4% and -8% respectively between 2013–2018 surveys) would mean reaching the SDG target of 25 under-five deaths per 1000 live births from the current U5MR of 64 per 1000 live births in another 10 to 15 years; rates of reduction must accelerate among the richer and urban groups to reach the target in that time. Further U5MR reductions across groups will require a particular focus on preventing mortality during the neonatal and increasingly intrapartum periods [27].

Overall inter-province differences in U5MR reduced absolutely but not relatively in this study, reflecting that some provinces have remained with higher levels. These were all farthest from the capital and bordering neighbour countries. One study estimating district-wise changes between 1990 and 2010 showed that U5MR reduced in all regions, but those starting at higher levels remained absolutely behind [34]. Those findings corresponded with our provincial estimates in 2013 ZDHS and earlier where Luapula, Northern, and Western provinces were worst off [34]. In 2018, we found that Muchinga (split from Northern in 2014) and Southern provinces also lagged, compared to Lusaka and Copperbelt. Another geospatial analysis of U5MR trends showed that Zambia’s district-wise variation was not as large as in many other Sub-Saharan Africa countries [1]. They further reported that U5MR reduced faster among districts starting with the highest levels, as much of the country reduced mortality faster than -5% per annum except for major urban areas of Lusaka (national capital) and Copperbelt (regional capital Ndola) provinces where baseline levels were already lower [1]. To build on this, our findings with double disaggregation suggest that provinces starting with greater wealth group inequalities also closed the gaps, like Northern, Central, Eastern and Western.

We also found that RMNCH intervention coverage inequalities declined consistently in Zambia over time. Earlier studies found that wealth quintile gaps in coverage were larger in Zambia compared to many sub-Saharan Africa countries in 2007 and 2013 ZDHS [22, 35, 36]. Our study’s analysis adding ZDHS 2018 showed that the CCI inequalities improved greatly in the most recent years as rates improved overall, whether using wealth quintiles and deciles, residence, and both wealth and residence. Gaps closed as the improvements were relatively fastest among the poorer and rural groups. CCI inequalities between provinces also reduced noticeably using both absolute and relative summary measures, similar to a previous study using ZDHS 2013, though we used a slight variation of CCI [37]. This was particularly true for provinces starting at lower baselines like Central, Luapula, Northern and Western. Though starting with wider intra-province wealth gaps, Luapula, Eastern and Western appeared to experience major CCI improvements among the poorest tertile as well.

Population health researchers have a growing set of equity measures to draw on for more granular and context-specific comparisons. Others have recently proposed using ethnicity as an equity stratifier, which is collected as self-reported ethnic affiliation in the DHS [24]. While its non-ordered nature may help in providing new insights for groups that are sufficiently large or meaningfully combined, the included groups have been found to vary across rounds and may not reflect consistent social hierarchies depending on a country’s cultural and historical context [24]. A range of other multi-dimensional measures of socio-economic position are also being developed, often composed of weighted indicators like education and living standards; not all suit the purpose of standardisation across countries, but rather should be adapted for specific socio-cultural, political and economic contexts [38]. It is valuable to consider and compare different measures of socio-economic position within a given context, as their components (education, assets or others) tend to improve unevenly and with different implications for health inequalities. For example, our related analyses showed that U5MR inequalities between relative wealth quintiles using asset indices reduced, but there were no accompanying improvements in absolute income levels, improved sanitation and water among poorer compared to richer wealth quintiles between 2001 and 2018, similar to a study in Kenya that compared asset-based and other measures of socio-economic status [27, 39].

Our study suggests that tracking health equity trends using survey data is valuable and can be taken further using a range of measures. Such analyses can also be built upon to pursue ever more nuanced understandings to guide targeting in national or subnational policies and programmes in a range of countries. Survey samples should be further increased to allow for multi-tier disaggregation, particularly for mortality inequalities, which is needed to track impact. Survey analyses using geospatial linkages with routinely-collected health facility data, which often include more process or readiness-related indicators, may advance assessments of equity in effective coverage that also link contact coverage to quality and health impacts [24, 40]. Socio-economic and spatial disadvantages tend to have compounding effects with respect to health inequities, which could be further examined through multilevel or decomposition regression modelling [41–44]. Future studies could also develop special surveys or qualitative studies to understand what societal and health systems factors combined to improve or perpetuate observed inequalities, such as in the most remote areas (like near the border, farther from the capital) or among disadvantaged socio-economic groups in urban settings of Zambia and other countries in the region [45–48].

This study had some limitations. Small samples for under-five mortality, even using all births in the ten-years preceding the surveys, made it harder to assess differences using wealth deciles or tertiles among provinces. While overall sampling expanded in 2013/14 and 2018 rounds of ZDHS, the numbers of deaths also decreased. Numbers of births were also higher among the poorer than richer groups, causing some groups to consistently have smaller samples. It was also not possible to directly compare asset indices used for wealth quintiles in urban versus rural areas given the way the DHS is designed, therefore we compared urban richest versus poorest and rural groups separately. Education is an important stratifier for health outcomes but showed similar patterns as wealth for the two outcomes we examined, and so are presented in supplementary materials. Also, trends and inequalities in U5MR and CCI estimates were not directly comparable as U5MR was measured among all births 10 years preceding survey to increase sample sizes, whereas CCI was among more recent births. This study did not include other interventions such as malaria prevention within the CCI that are also relevant to U5MR, and which we included in our analyses elsewhere; we used the standard CCI for comparability to related studies [27]. Future research could also consider the role of food security or nutrition indicators in understanding child health inequalities.

## Conclusion

This study examined trends in RMNCH inequalities using the last four rounds of the Zambia Demographic Health Survey to consider how much more surveys can reveal about who experienced the greatest improvements or have been left behind. We found that multi-tier equity measures with expanded categorisations of socio-economic (wealth) and spatial (residence or province) stratifiers in many cases gave plausible and acceptable estimates that provided new insights over standard measures, particularly for the composite coverage index and when using non-ordered groups like provinces. Visualisations such as equiplots helped assess who have become most left behind, even with less certain estimates, while summary measures aided in assessing inequalities across populations. Sample sizes in future surveys must be enlarged to account for further disaggregation if we are to reliably capture inequality trends in outcomes like mortality. Lessons learned in this study can inform future health equity research in a range of contexts seeking to understand intersecting disadvantages that compound poor health outcomes and within-group differences. In these ways, survey analyses using multi-tier equity measures, combined with health facility data and qualitative research, would helpfully guide efforts for improving effective coverage for RMNCH that truly leave no woman or child behind.

## Supplementary Information

**Additional file 1:** **Supplementary Table 1.** Under-five mortality rates (per1000 live births) by different stratifiers (ten years preceding the survey) with 95% confidence intervals, ZDHS 2001/2, 2007, 2013/14, 2018. **Supplementary Table 2.** Composite coverage index (CCI, %) for RMNCH interventions by different stratifiers with 95% confidence intervals, ZDHS 2001/1, 2007, 2013/14, 2018. **Supplementary Table 3.** Under-five mortality rates (per 1000 live births) by province and wealthtertile with 95% confidence intervals, ZDHS 2001/2, 2007, 2013/14, 2018. **Supplementary Table 4.** Composite coverage index (CCI, %) by province and wealth tertile with 95% confidence intervals, ZDHS 2001/2, 2007, 2013/14, 2018. **Supplementary Table 5.** Precision assessment using percentage difference between confidence interval and mean estimate, divided by mean estimate (%). 

## Data Availability

Data is publicly available from the DHS program website upon request.

## Abbreviations

ANC4: Antenatal care four or more visits
BCG: Bacille Calmette-Guerin
DPT3: Diphtheria-Pertussis-Tetanus vaccination three doses
CAREANYD: Care-seeking for any childhood illness
CCI: Composite Coverage Index
CI: Confidence Interval
CIX: Concentration Index
D1-D10: Decile 1‐10
DHS: Demographic Health Survey
FPCmo: Demand for family planning satisfied with modern methods
MSL: Measles vaccination
Q1-Q5: Quintile 1‐5
RMNCH: Reproductive, Maternal, Newborn and Child Health
SBA: Skilled Birth Attendance
SDG: Sustainable Development Goal
SII: Slope Index of Inequality
SE: Standard Error
T1-T3: Tertile 1‐3
U5MR: Under-five Mortality Rate
WMDM: Weighted Mean Difference from Overall Mean
ZDHS: Zambia Demographic and Health Survey
