# Supplementary material for: Going deeper with health equity measurement: how much more can surveys reveal about inequalities in health intervention coverage and mortality in Zambia?
Source: Int J Equity Health. 2023 Jun 2;22:109. doi: 10.1186/s12939-023-01901-x (PMC10236645; doi:10.1186/s12939-023-01901-x)
Supplement: Supplementary file 1 — Additional file 1: Supplementary Table 1. Under-five mortality rates (per1000 live births) by different stratifiers (ten years preceding the survey) with 95% confidence intervals, ZDHS 2001/2, 2007, 2013/14, 2018. Supplementary Table 2. Composite coverage index (CCI, %) for RMNCH interventions by different stratifiers with 95% confidence intervals, ZDHS 2001/1, 2007, 2013/14, 2018. Supplementary Table 3. Under-five mortality rates (per 1000 live births) by province and wealthtertile with 95% confidence intervals, ZDHS 2001/2, 2007, 2013/14, 2018. Supplementary Table 4. Composite coverage index (CCI, %) by province and wealth tertile with 95% confidence intervals, ZDHS 2001/2, 2007, 2013/14, 2018. Supplementary Table 5. Precision assessment using percentage difference between confidence interval and mean estimate, divided by mean estimate (%). [file 12939_2023_1901_MOESM1_ESM.docx]

**Supplementary materials**

Supplementary Table 1: Under-five mortality rates (per 1000 live births) by different stratifiers (ten years preceding the survey) with 95% confidence intervals, ZDHS 2001/2, 2007, 2013/14, 2018

|  | **2001/2** | | | **2007** | | | **2013/14** | | | **2018** | | |
| --- | --- | --- | --- | --- | --- | --- | --- | --- | --- | --- | --- | --- |
|  | **U5MR** | **95% CI lower bound** | **95% CI upper bound** | **U5MR** | **95% CI lower bound** | **95% CI upper bound** | **U5MR** | **95% CI lower bound** | **95% CI upper bound** | **U5MR** | **95% CI lower bound** | **95% CI upper bound** |
| **National** | 167.94 | 161.04 | 174.84 | 136.85 | 129.92 | 143.77 | 80.63 | 76.73 | 84.53 | 64.27 | 59.60 | 68.94 |
| **Province** |  |  |  |  |  |  |  |  |  |  |  |  |
| Central | 192.06 | 171.65 | 212.48 | 117.55 | 97.82 | 137.29 | 79.90 | 67.56 | 92.24 | 47.14 | 37.35 | 56.92 |
| Copperbelt | 133.68 | 115.57 | 151.78 | 133.08 | 112.54 | 153.62 | 63.19 | 52.52 | 73.87 | 49.57 | 38.06 | 61.08 |
| Eastern | 166.13 | 147.33 | 184.94 | 151.02 | 132.57 | 169.47 | 114.53 | 102.19 | 126.87 | 63.96 | 53.47 | 74.45 |
| Luapula | 248.47 | 222.42 | 274.52 | 156.96 | 136.12 | 177.81 | 97.63 | 85.95 | 109.31 | 110.28 | 96.31 | 124.25 |
| Lusaka | 137.04 | 117.65 | 156.42 | 135.04 | 113.79 | 156.29 | 68.50 | 56.77 | 80.22 | 63.92 | 51.90 | 75.93 |
| Muchinga | NA | NA | NA | NA | NA | NA | 88.32 | 75.13 | 101.51 | 74.91 | 62.40 | 87.42 |
| Northern | 187.17 | 170.09 | 204.25 | 159.41 | 138.88 | 179.93 | 86.11 | 74.43 | 97.80 | 65.54 | 53.64 | 77.43 |
| North Western | 129.95 | 113.25 | 146.66 | 107.87 | 89.51 | 126.22 | 66.14 | 55.49 | 76.78 | 26.08 | 16.70 | 35.46 |
| Southern | 147.55 | 127.01 | 168.09 | 102.97 | 86.09 | 119.86 | 68.11 | 57.46 | 78.76 | 70.18 | 47.47 | 92.89 |
| Western | 201.10 | 176.89 | 225.31 | 139.47 | 117.91 | 161.02 | 72.68 | 58.81 | 86.54 | 57.38 | 45.47 | 69.29 |
| **Residence** |  |  |  |  |  |  |  |  |  |  |  |  |
| Rural | 182.26 | 173.84 | 190.69 | 138.87 | 130.66 | 147.09 | 85.06 | 80.28 | 89.85 | 61.90 | 57.44 | 66.36 |
| Urban | 140.03 | 128.07 | 151.99 | 132.20 | 119.34 | 145.07 | 72.27 | 65.56 | 78.98 | 68.46 | 58.26 | 78.66 |
| **Wealth (Overall)** |  |  |  |  |  |  |  |  |  |  |  |  |
| Q1 | 191.67 | 176.88 | 206.46 | 123.76 | 109.74 | 137.78 | 99.53 | 90.89 | 108.18 | 66.56 | 59.36 | 73.76 |
| Q2 | 182.54 | 167.06 | 198.03 | 147.74 | 133.13 | 162.35 | 84.58 | 76.69 | 92.48 | 66.88 | 58.88 | 74.88 |
| Q3 | 196.08 | 180.80 | 211.37 | 155.46 | 139.97 | 170.94 | 78.92 | 71.04 | 86.80 | 52.64 | 45.07 | 60.20 |
| Q4 | 163.22 | 146.84 | 179.60 | 140.22 | 124.04 | 156.39 | 72.98 | 63.22 | 82.73 | 76.23 | 59.79 | 92.68 |
| Q5 | 92.44 | 77.89 | 106.99 | 110.21 | 92.30 | 128.11 | 57.86 | 48.18 | 67.54 | 57.41 | 45.24 | 69.57 |
| **Mother's Education** |  |  |  |  |  |  |  |  |  |  |  |  |
| No education | 197.76 | 179.12 | 216.41 | 143.87 | 125.53 | 162.21 | 108.98 | 96.56 | 121.41 | 69.22 | 58.33 | 80.10 |
| Primary | 177.21 | 168.41 | 186.01 | 145.89 | 136.95 | 154.83 | 82.15 | 77.05 | 87.24 | 65.54 | 59.14 | 71.94 |
| Secondary | 122.98 | 108.79 | 137.16 | 104.66 | 90.92 | 118.40 | 66.75 | 59.52 | 73.98 | 61.71 | 52.55 | 70.87 |
| Higher | 102.03 | 56.42 | 147.65 | 109.00 | 65.87 | 152.13 | 42.81 | 26.32 | 59.30 | 46.50 | 28.69 | 64.31 |
| **Wealth (rural)** |  |  |  |  |  |  |  |  |  |  |  |  |
| Q1 - rural | 207.84 | 188.69 | 226.98 | 107.78 | 90.84 | 124.71 | 105.70 | 94.33 | 117.08 | 67.22 | 58.17 | 76.28 |
| Q2 - rural | 175.16 | 156.08 | 194.25 | 146.47 | 127.56 | 165.39 | 89.09 | 78.33 | 99.85 | 67.05 | 57.29 | 76.82 |
| Q3 - rural | 174.73 | 156.50 | 192.96 | 148.47 | 129.89 | 167.05 | 81.30 | 70.96 | 91.64 | 63.95 | 53.64 | 74.26 |
| Q4 - rural | 185.80 | 167.22 | 204.38 | 159.32 | 139.64 | 178.99 | 83.24 | 72.44 | 94.04 | 61.39 | 50.83 | 71.95 |
| Q5 - rural | 164.86 | 145.87 | 183.85 | 132.80 | 115.23 | 150.36 | 63.56 | 53.58 | 73.54 | 45.29 | 35.02 | 55.55 |
| **Wealth (Urban)** |  |  |  |  |  |  |  |  |  |  |  |  |
| Q1 - urban | 198.84 | 169.52 | 228.15 | 172.48 | 143.72 | 201.23 | 91.15 | 78.48 | 103.81 | 56.90 | 45.41 | 68.40 |
| Q2 - urban | 179.06 | 150.27 | 207.85 | 160.69 | 129.77 | 191.62 | 78.64 | 62.58 | 94.71 | 92.28 | 58.64 | 12.59 |
| Q3 - urban | 143.37 | 114.89 | 171.84 | 118.48 | 90.61 | 146.36 | 71.22 | 54.37 | 88.08 | 74.97 | 52.87 | 97.08 |
| Q4 - urban | 95.59 | 72.11 | 119.08 | 94.60 | 68.27 | 120.92 | 57.18 | 42.70 | 71.66 | 50.34 | 35.20 | 65.49 |
| Q5 - urban | 88.89 | 65.74 | 112.04 | 112.79 | 83.39 | 142.19 | 61.75 | 46.96 | 76.54 | 62.78 | 43.92 | 81.64 |
| **Wealth decile** |  |  |  |  |  |  |  |  |  |  |  |  |
| D1 | 213.81 | 191.07 | 236.55 | 111.55 | 88.06 | 135.04 | 99.97 | 85.99 | 113.96 | 67.50 | 55.54 | 79.47 |
| D2 | 158.69 | 136.60 | 180.77 | 138.28 | 116.56 | 159.99 | 99.11 | 82.21 | 116.00 | 65.38 | 53.24 | 77.52 |
| D3 | 182.13 | 158.13 | 206.12 | 137.97 | 116.10 | 159.85 | 92.31 | 78.73 | 105.89 | 71.72 | 55.26 | 88.19 |
| D4 | 182.89 | 161.58 | 204.20 | 155.64 | 132.98 | 178.30 | 76.76 | 63.84 | 89.68 | 62.04 | 51.33 | 72.75 |
| D5 | 181.22 | 156.92 | 205.51 | 161.03 | 133.90 | 188.17 | 84.22 | 72.10 | 96.33 | 57.40 | 44.24 | 70.55 |
| D6 | 212.36 | 185.95 | 238.77 | 150.62 | 127.87 | 173.38 | 73.55 | 60.98 | 86.13 | 47.65 | 34.66 | 60.64 |
| D7 | 175.46 | 152.02 | 198.89 | 146.15 | 118.81 | 173.49 | 75.47 | 61.61 | 89.33 | 90.86 | 28.68 | 153.03 |
| D8 | 149.60 | 123.14 | 176.06 | 134.07 | 105.50 | 162.64 | 70.20 | 54.83 | 85.57 | 62.16 | 41.21 | 83.11 |
| D9 | 96.53 | 74.12 | 118.94 | 110.10 | 90.06 | 130.14 | 55.27 | 40.79 | 69.75 | 55.01 | 37.30 | 72.72 |
| D10 | 87.90 | 59.45 | 116.36 | 110.33 | 85.35 | 135.30 | 60.88 | 41.79 | 79.96 | 60.31 | 41.33 | 79.29 |

Supplementary Table 2: Composite coverage index (CCI, %) for RMNCH interventions by different stratifiers with 95% confidence intervals, ZDHS 2001/1, 2007, 2013/14, 2018

|  | **2001/2** | | | **2007** | | | **2013/14** | | | **2018** | | |
| --- | --- | --- | --- | --- | --- | --- | --- | --- | --- | --- | --- | --- |
|  | **CCI** | **95% CI lower bound** | **95% CI upper bound** | **CCI** | **95% CI lower bound** | **95% CI upper bound** | **CCI** | **95% CI lower bound** | **95% CI upper bound** | **CCI** | **95% CI lower bound** | **95% CI upper bound** |
| **National** | 60.06 | 59.12 | 61.01 | 59.70 | 58.76 | 60.64 | 70.06 | 69.42 | 70.69 | 76.20 | 75.30 | 77.09 |
| **Province** |  |  |  |  |  |  |  |  |  |  |  |  |
| Central | 56.23 | 54.20 | 58.26 | 58.95 | 56.13 | 61.78 | 63.49 | 60.83 | 66.15 | 76.20 | 73.71 | 78.68 |
| Copperbelt | 69.74 | 67.30 | 72.19 | 65.99 | 62.53 | 69.44 | 75.78 | 73.88 | 77.69 | 78.06 | 75.75 | 80.37 |
| Eastern | 60.27 | 58.00 | 62.53 | 61.55 | 59.34 | 63.76 | 73.52 | 71.79 | 75.24 | 80.91 | 79.41 | 82.40 |
| Luapula | 52.45 | 49.65 | 55.24 | 50.06 | 46.56 | 53.56 | 66.67 | 64.72 | 68.61 | 74.95 | 72.65 | 77.26 |
| Lusaka | 70.28 | 68.01 | 72.55 | 67.58 | 64.37 | 70.80 | 76.56 | 74.78 | 78.34 | 76.09 | 73.58 | 78.61 |
| Muchinga | NA | NA | NA | NA | NA | NA | 63.37 | 61.07 | 65.68 | 74.99 | 72.31 | 77.67 |
| North Western | 60.37 | 57.78 | 62.95 | 57.13 | 54.48 | 59.79 | 71.92 | 69.94 | 73.90 | 78.12 | 75.53 | 80.70 |
| Northern | 50.26 | 48.58 | 51.94 | 48.60 | 45.50 | 51.70 | 61.96 | 59.80 | 64.13 | 70.53 | 67.99 | 73.07 |
| Southern | 58.63 | 56.04 | 61.21 | 65.45 | 63.47 | 67.44 | 69.62 | 67.85 | 71.39 | 73.99 | 71.05 | 76.93 |
| Western | 50.96 | 48.30 | 53.61 | 60.76 | 57.54 | 63.97 | 64.30 | 61.93 | 66.66 | 69.79 | 67.40 | 72.17 |
| **Area** |  |  |  |  |  |  |  |  |  |  |  |  |
| Rural | 54.23 | 53.29 | 55.18 | 55.15 | 54.22 | 56.08 | 66.10 | 65.36 | 66.84 | 75.20 | 74.30 | 76.11 |
| Urban | 70.88 | 69.25 | 72.52 | 69.09 | 67.25 | 70.94 | 76.90 | 75.59 | 78.21 | 77.54 | 75.75 | 79.33 |
| **Education** |  |  |  |  |  |  |  |  |  |  |  |  |
| No education | 46.32 | 44.50 | 48.15 | 50.25 | 47.66 | 52.84 | 60.94 | 58.76 | 63.13 | 67.44 | 65.26 | 69.64 |
| Primary | 58.18 | 57.11 | 59.25 | 57.50 | 56.51 | 58.49 | 68.11 | 67.24 | 68.97 | 75.02 | 74.05 | 76.00 |
| Secondary | 71.98 | 69.97 | 74.00 | 68.48 | 66.53 | 70.42 | 75.07 | 73.93 | 76.22 | 79.75 | 78.43 | 81.07 |
| Higher | 79.18 | 73.12 | 85.24 | 80.75 | 73.93 | 87.58 | 83.27 | 80.27 | 86.27 | 80.81 | 77.33 | 84.28 |
| **Wealth quintile** |  |  |  |  |  |  |  |  |  |  |  |  |
| Q1 | 48.78 | 47.47 | 50.08 | 53.59 | 51.56 | 55.62 | 61.97 | 60.53 | 63.41 | 72.31 | 70.90 | 73.73 |
| Q2 | 52.38 | 50.74 | 54.01 | 53.43 | 51.71 | 55.16 | 66.28 | 65.04 | 67.52 | 75.11 | 73.62 | 76.59 |
| Q3 | 56.10 | 54.31 | 57.90 | 54.44 | 52.60 | 56.29 | 69.49 | 68.15 | 70.82 | 77.38 | 75.14 | 79.62 |
| Q4 | 67.78 | 66.39 | 69.16 | 66.72 | 64.64 | 68.79 | 75.36 | 73.61 | 77.10 | 77.05 | 74.72 | 79.37 |
| Q5 | 76.33 | 74.08 | 78.58 | 73.33 | 71.17 | 75.49 | 80.12 | 78.67 | 81.58 | 79.40 | 76.81 | 82.98 |
| **Wealth quintile (rural)** |  |  |  |  |  |  |  |  |  |  |  |  |
| Q1 | 47.75 | 45.91 | 49.60 | 52.25 | 49.86 | 54.63 | 61. 73 | 60.14 | 63.32 | 69.09 | 67.24 | 70.93 |
| Q2 | 49.93 | 48.02 | 51.83 | 55.26 | 52.99 | 57.53 | 62.96 | 61.22 | 64.70 | 76.49 | 74.59 | 78.38 |
| Q3 | 54.11 | 51.74 | 56.47 | 52.80 | 50.30 | 55.31 | 65.82 | 64.34 | 67.30 | 75.36 | 73.67 | 77.06 |
| Q4 | 54.48 | 52.03 | 56.94 | 54.42 | 51.89 | 56.95 | 68.25 | 66.42 | 70.09 | 76.57 | 74.63 | 78.50 |
| Q5 | 64.43 | 62.14 | 66.72 | 61.58 | 58.90 | 64.25 | 71.80 | 69.65 | 73.95 | 78.53 | 76.21 | 80.84 |
| **Wealth quintile (urban)** |  |  |  |  |  |  |  |  |  |  |  |  |
| Q1 | 59.46 | 56.14 | 62.78 | 61.99 | 59.07 | 64.90 | 72.91 | 71.05 | 74.78 | 76.92 | 73.73 | 80.11 |
| Q2 | 66.66 | 63.40 | 69.93 | 65.93 | 62.05 | 69.81 | 74.38 | 71.87 | 76.88 | 75.76 | 71.95 | 79.58 |
| Q3 | 75.92 | 72.52 | 79.33 | 70.78 | 66.96 | 74.60 | 77.46 | 74.72 | 80.20 | 76.36 | 72.89 | 79.83 |
| Q4 | 71.99 | 67.26 | 76.72 | 73.03 | 68.65 | 77.40 | 80.33 | 77.82 | 82.85 | 79.79 | 76.10 | 83.48 |
| Q5 | 81.70 | 77.95 | 85.44 | 75.06 | 70.83 | 79.29 | 80.45 | 77.67 | 83.23 | 79.37 | 75.86 | 82.89 |
| **Wealth decile** |  |  |  |  |  |  |  |  |  |  |  |  |
| D1 | 46.32 | 44.16 | 48.47 | 50.92 | 48.64 | 53.20 | 61.47 | 59.51 | 63.42 | 69.11 | 66.83 | 71.38 |
| D2 | 52.35 | 50.06 | 54.63 | 56.61 | 54.20 | 59.02 | 62.39 | 60.26 | 64.51 | 75.75 | 73.85 | 77.65 |
| D3 | 51.43 | 48.99 | 53.86 | 52.32 | 49.35 | 55.29 | 65.53 | 63.20 | 67.86 | 74.70 | 72.72 | 76.69 |
| D4 | 53.04 | 50.70 | 55.38 | 54.47 | 51.26 | 57.68 | 67.01 | 65.11 | 68.90 | 75.47 | 73.38 | 77.55 |
| D5 | 54.36 | 52.10 | 56.62 | 53.70 | 50.51 | 56.89 | 69.48 | 67.55 | 71.42 | 77.86 | 75.80 | 79.92 |
| D6 | 57.89 | 55.37 | 60.42 | 55.38 | 52.71 | 58.05 | 69.49 | 66.84 | 72.15 | 76.72 | 73.59 | 79.84 |
| D7 | 63.27 | 60.70 | 65.84 | 64.74 | 61.72 | 67.76 | 74.82 | 73.06 | 76.57 | 77.69 | 74.80 | 80.58 |
| D8 | 72.35 | 69.56 | 75.14 | 68.75 | 65.72 | 71.79 | 76.33 | 73.85 | 78.82 | 76.31 | 72.65 | 79.96 |
| D9 | 73.25 | 70.19 | 76.31 | 71.51 | 67.97 | 75.04 | 79.57 | 77.36 | 81.79 | 78.84 | 75.68 | 81.99 |
| D10 | 81.06 | 78.02 | 84.10 | 74.98 | 71.98 | 77.99 | 80.60 | 77.92 | 83.29 | 80.34 | 77.17 | 83.50 |

Supplementary Table 3: Under-five mortality rates (per 1000 live births) by province and wealth tertile with 95% confidence intervals, ZDHS 2001/2, 2007, 2013/14, 2018

|  |  | **2001/2** | | | **2007** | | | **2013/14** | | | **2018** | | |
| --- | --- | --- | --- | --- | --- | --- | --- | --- | --- | --- | --- | --- | --- |
| **Province** | **Wealth tertile** | **U5MR** | **95% CI lower bound** | **95% CI upper bound** | **U5MR** | **95% CI lower bound** | **95% CI upper bound** | **U5MR** | **95% CI lower bound** | **95% CI upper bound** | **U5MR** | **95% CI lower bound** | **95% CI upper bound** |
| Central | T1 | 208.67 | 172.00 | 245.35 | 138.37 | 95.97 | 180.78 | 96.79 | 62.21 | 131.38 | 57.04 | 35.98 | 78.10 |
|  | T2 | 198.16 | 142.56 | 253.75 | 113.83 | 83.91 | 143.75 | 77.06 | 59.85 | 94.27 | 38.13 | 21.35 | 54.91 |
|  | T3 | 147.64 | 110.55 | 184.74 | 77.48 | 42.55 | 112.42 | 53.19 | 30.96 | 75.43 | 50.94 | 15.63 | 86.25 |
| Copperbelt | T1 | 155.27 | 65.02 | 245.52 | 109.85 | 39.52 | 180.18 | 97.83 | 63.54 | 132.12 | 54.70 | 19.44 | 89.97 |
|  | T2 | 201.36 | 160.49 | 242.24 | 180.91 | 134.68 | 227.15 | 69.56 | 46.86 | 92.26 | 63.11 | 40.39 | 85.82 |
|  | T3 | 107.39 | 78.74 | 136.05 | 118.35 | 94.13 | 142.58 | 53.72 | 35.70 | 71.74 | 41.55 | 27.45 | 55.64 |
| Eastern | T1 | 191.17 | 156.41 | 225.93 | 139.65 | 112.33 | 166.96 | 122.74 | 103.54 | 141.94 | 66.71 | 51.27 | 82.16 |
|  | T2 | 141.42 | 108.14 | 174.69 | 185.64 | 145.11 | 226.17 | 104.53 | 83.38 | 125.69 | 62.55 | 44.51 | 80.60 |
|  | T3 | 137.82 | 82.99 | 192.65 | 116.91 | 57.14 | 176.69 | 106.07 | 58.64 | 153.51 | 49.53 | 14.25 | 84.81 |
| Luapula | T1 | 218.81 | 170.20 | 267.41 | 159.83 | 108.70 | 210.96 | 106.72 | 74.99 | 138.44 | 113.28 | 87.71 | 138.85 |
|  | T2 | 267.84 | 226.13 | 309.56 | 152.08 | 115.92 | 188.24 | 86.63 | 62.95 | 110.32 | 109.69 | 83.67 | 135.70 |
|  | T3 | 198.00 | 96.70 | 299.31 | 195.37 | 105.01 | 285.72 | 72.71 | 40.95 | 104.47 | 90.60 | 40.84 | 140.35 |
| Lusaka | T1 | 174.23 | -5.96 | 354.43 | 136.53 | 74.49 | 198.57 | 58.79 | 7.59 | 109.98 | 31.96 | -3.93 | 67.86 |
|  | T2 | 178.14 | 129.69 | 226.59 | 101.36 | 57.88 | 144.85 | 82.81 | 48.05 | 117.58 | 41.71 | 15.90 | 67.52 |
|  | T3 | 130.49 | 104.36 | 156.61 | 140.26 | 115.02 | 165.51 | 65.01 | 49.69 | 80.34 | 70.32 | 49.86 | 90.79 |
| Muchinga | T1 | NA | NA | NA | NA | NA | NA | 101.82 | 81.93 | 121.71 | 84.25 | 60.52 | 107.99 |
|  | T2 | NA | NA | NA | NA | NA | NA | 72.92 | 53.89 | 91.95 | 63.20 | 41.98 | 84.43 |
|  | T3 | NA | NA | NA | NA | NA | NA | 57.08 | 30.09 | 84.06 | 48.28 | 10.29 | 86.26 |
| North-Western | T1 | 120.74 | 99.21 | 142.26 | 103.33 | 77.51 | 129.15 | 61.14 | 42.56 | 79.72 | 27.06 | 16.16 | 37.95 |
|  | T2 | 143.24 | 108.90 | 177.58 | 113.82 | 72.27 | 155.38 | 70.70 | 51.19 | 90.21 | 24.98 | 8.84 | 41.11 |
|  | T3 | 121.45 | 38.55 | 204.34 | 104.08 | 40.75 | 167.41 |  |  |  |  |  |  |
| Northern | T1 | 203.87 | 175.94 | 231.80 | 166.32 | 134.40 | 198.24 | 91.26 | 71.94 | 110.57 | 60.37 | 36.75 | 83.99 |
|  | T2 | 186.57 | 153.87 | 219.27 | 160.43 | 119.85 | 201.02 | 81.07 | 61.37 | 100.78 | 72.22 | 30.73 | 113.71 |
|  | T3 | 119.35 | 71.66 | 167.04 | 119.91 | 86.25 | 153.57 | 60.55 | 24.79 | 96.31 | 85.63 | 44.19 | 127.07 |
| Southern | T1 | 137.89 | 106.63 | 169.16 | 77.17 | 47.66 | 106.68 | 67.10 | 42.78 | 91.42 | 47.43 | 27.57 | 67.28 |
|  | T2 | 161.65 | 121.55 | 201.75 | 113.48 | 86.11 | 140.85 | 66.03 | 50.96 | 81.11 | 50.22 | 33.44 | 67.01 |
|  | T3 | 135.32 | 48.82 | 221.82 | 124.05 | 79.62 | 168.48 |  |  |  |  |  |  |
| Western | T1 | 212.09 | 174.80 | 249.38 | 131.26 | 93.35 | 169.17 | 73.36 | 37.47 | 109.25 | 52.75 | 35.93 | 69.57 |
|  | T2 | 172.18 | 104.70 | 239.66 | 175.27 | 121.56 | 228.98 | 76.62 | 49.03 | 104.20 | 73.19 | 44.44 | 101.95 |
|  | T3 | 158.95 | 116.34 | 201.56 | 88.46 | -4.12 | 181.05 |  |  |  |  |  |  |

Supplementary Table 4: Composite coverage index (CCI, %) by province and wealth tertile with 95% confidence intervals, ZDHS 2001/2, 2007, 2013/14, 2018

|  |  | **2001/2** | | | **2007** | | | **2013/14** | | | **2018** | | |
| --- | --- | --- | --- | --- | --- | --- | --- | --- | --- | --- | --- | --- | --- |
| **Province** | **Wealth tertile** | **CCI** | **95% CI lower bound** | **95% CI upper bound** | **CCI** | **95% CI lower bound** | **95% CI upper bound** | **CCI** | **95% CI lower bound** | **95% CI upper bound** | **CCI** | **95% CI lower bound** | **95% CI upper bound** |
| Central | T1 | 46.36 | 41.44 | 51.27 | 51.19 | 43.63 | 58.74 | 58.06 | 51.41 | 64.72 | 71.04 | 65.87 | 76.22 |
|  | T2 | 54.88 | 50.37 | 59.40 | 58.48 | 52.23 | 64.73 | 64.95 | 59.28 | 70.61 |  |  |  |
|  | T3 |  |  |  | 76.56 | 70.07 | 83.05 | 75.60 | 68.14 | 83.06 | 81.52 | 78.35 | 84.68 |
| Copperbelt | T1 |  |  |  | 62.85 | 55.70 | 70.00 | 64.83 | 57.89 | 71.76 |  |  |  |
|  | T2 | 59.89 | 53.72 | 66.07 | 56.65 | 45.63 | 67.67 |  |  |  | 78.01 | 73.95 | 82.06 |
|  | T3 | 74.15 | 70.98 | 77.32 | 68.36 | 64.31 | 72.41 | 79.61 | 75.09 | 84.13 | 77.68 | 75.26 | 80.11 |
| Eastern | T1 | 54.27 | 50.79 | 57.75 | 57.86 | 54.82 | 60.90 | 72.81 | 69.88 | 75.73 | 80.51 | 76.72 | 84.30 |
|  | T2 | 61.31 | 58.34 | 64.27 | 65.86 | 61.41 | 70.32 | 74.09 | 70.79 | 77.39 | 81.35 | 79.30 | 83.39 |
|  | T3 | 79.97 | 75.05 | 84.88 | 74.70 | 68.37 | 81.03 |  |  |  |  |  |  |
| Luapula | T1 | 49.11 | 40.40 | 57.83 | 46.82 | 34.64 | 58.99 | 63.35 | 58.05 | 68.66 | 72.17 | 67.34 | 77.00 |
|  | T2 | 51.34 | 47.44 | 55.23 | 49.05 | 44.47 | 53.64 | 70.69 | 64.45 | 76.94 | 76.78 | 71.55 | 82.01 |
|  | T3 | 68.31 | 59.04 | 77.58 | 75.22 | 69.04 | 81.41 | 81.14 | 77.15 | 85.14 |  |  |  |
| Lusaka | T1 |  |  |  | 63.78 | 55.39 | 72.16 | 77.32 | 69.56 | 85.07 |  |  |  |
|  | T2 | 56.22 | 49.00 | 63.44 | 57.06 | 50.47 | 63.65 | 71.51 | 65.07 | 77.96 | 80.21 | 76.77 | 83.65 |
|  | T3 | 72.84 | 70.92 | 74.75 | 68.65 | 64.39 | 72.90 | 78.24 | 74.20 | 82.28 | 75.70 | 73.61 | 77.79 |
| Muchinga | T1 | NA | NA | NA | NA | NA | NA | 59.84 | 55.24 | 64.45 | 72.00 | 65.93 | 78.08 |
|  | T2 | NA | NA | NA | NA | NA | NA | 66.49 | 60.17 | 72.81 | 79.11 | 73.79 | 84.43 |
|  | T3 | NA | NA | NA | NA | NA | NA | 72.98 | 68.03 | 77.93 |  |  |  |
| North-Western | T1 | 58.19 | 52.92 | 63.46 | 56.58 | 49.43 | 63.72 | 67.68 | 64.77 | 70.59 | 79.11 | 74.47 | 83.76 |
|  | T2 | 59.41 | 54.67 | 64.16 | 59.10 | 52.40 | 65.81 | 74.37 | 70.24 | 78.50 |  |  |  |
|  | T3 |  |  |  | 68.18 | 61.15 | 75.20 | 80.30 | 74.19 | 86.41 |  |  |  |
| Northern | T1 | 43.79 | 39.84 | 47.74 | 45.70 | 40.81 | 50.59 | 56.78 | 51.13 | 62.43 | 67.65 | 62.46 | 72.84 |
|  | T2 | 51.34 | 47.35 | 55.32 | 50.02 | 43.48 | 56.57 | 70.10 | 66.78 | 73.41 |  |  |  |
|  | T3 |  |  |  | 70.17 | 64.86 | 75.47 |  |  |  |  |  |  |
| Southern | T1 | 54.38 | 49.20 | 59.57 | 56.35 | 52.56 | 60.13 | 65.73 | 60.66 | 70.80 | 70.41 | 65.08 | 75.73 |
|  | T2 | 58.68 | 53.93 | 63.42 | 63.10 | 58.88 | 67.32 | 68.02 | 64.46 | 71.59 | 72.65 | 67.76 | 77.53 |
|  | T3 |  |  |  | 83.77 | 80.03 | 87.52 | 81.61 | 77.64 | 85.58 |  |  |  |
| Western | T1 | 46.57 | 41.45 | 51.68 | 58.41 | 52.11 | 64.71 | 61.20 | 55.36 | 67.03 | 67.11 | 60.92 | 73.31 |
|  | T2 | 64.79 | 57.42 | 72.17 |  |  |  | 71.35 | 67.32 | 75.38 | 72.59 | 66.03 | 79.16 |
|  | T3 |  |  |  |  |  |  | 78.38 | 75.12 | 81.64 | 78.22 | 73.28 | 83.16 |

Supplementary Table 5: Precision assessment using percentage difference between confidence interval and mean estimate, divided by mean estimate (%)

|  |  | **Under-five mortality rate** | | | | **Composite coverage index** | | | |
| --- | --- | --- | --- | --- | --- | --- | --- | --- | --- |
| **Stratifier** | **Category** | **2001/2** | **2007** | **2013/14** | **2018** | **2001/2** | **2007** | **2013/14** | **2018** |
| **National** |  | 4.1 | 5.1 | 4.8 | 7.3 | 1.6 | 1.6 | 0.9 | 1.2 |
| **Residence** | Rural | 4.6 | 5.9 | 5.6 | 7.2 | 1.7 | 1.7 | 1.1 | 1.2 |
|  | Urban | 8.5 | 9.7 | 9.3 | 14.9 | 2.3 | 2.7 | 1.7 | 2.3 |
| **Province** | Central | 10.6 | 16.8 | 15.4 | 20.8 | 3.6 | 4.8 | 4.2 | 3.3 |
|  | Copperbelt | 13.5 | 15.4 | 16.9 | 23.2 | 3.5 | 5.2 | 2.5 | 3.0 |
|  | Eastern | 11.3 | 12.2 | 10.8 | 16.4 | 3.8 | 3.6 | 2.4 | 1.9 |
|  | Luapula | 10.5 | 13.3 | 12.0 | 12.7 | 5.3 | 7.0 | 2.9 | 3.1 |
|  | Lusaka | 14.1 | 15.7 | 17.1 | 18.8 | 3.2 | 4.7 | 2.3 | 3.3 |
|  | Muchinga | NA | NA | 14.9 | 16.7 | NA | NA | 3.6 | 3.6 |
|  | North Western | 9.1 | 12.9 | 13.6 | 18.2 | 4.3 | 4.6 | 2.8 | 3.3 |
|  | Northern | 12.9 | 17.0 | 16.1 | 36.0 | 3.3 | 6.4 | 3.5 | 3.6 |
|  | Southern | 13.9 | 16.4 | 15.6 | 32.4 | 4.4 | 3.0 | 2.5 | 4.0 |
|  | Western | 12.0 | 15.5 | 19.1 | 20.8 | 5.2 | 5.3 | 3.7 | 3.4 |
| **Wealth quintile** | Q1 | 7.7 | 11.3 | 8.7 | 10.8 | 2.7 | 3.8 | 2.3 | 1.9 |
|  | Q2 | 8.5 | 9.9 | 9.3 | 12.0 | 3.1 | 3.2 | 1.9 | 2.0 |
|  | Q3 | 7.8 | 10.0 | 10.0 | 14.4 | 3.2 | 3.4 | 1.9 | 2.9 |
|  | Q4 | 10.0 | 11.5 | 13.4 | 21.6 | 2.1 | 3.1 | 2.3 | 3.0 |
|  | Q5 | 15.7 | 16.3 | 16.7 | 21.2 | 2.9 | 2.9 | 1.8 | 3.3 |
| **Wealth - urban** | Q1 - urban | 14.7 | 16.7 | 13.9 | 20.2 | 5.6 | 4.7 | 2.6 | 4.1 |
|  | Q2 - urban | 16.1 | 19.2 | 20.4 | 36.5 | 4.9 | 5.9 | 3.4 | 5.0 |
|  | Q3 - urban | 19.9 | 23.5 | 23.7 | 29.5 | 4.5 | 5.4 | 3.5 | 4.5 |
|  | Q4 - urban | 24.6 | 27.8 | 25.3 | 30.1 | 6.6 | 6.0 | 3.1 | 4.6 |
|  | Q5 - urban | 26.0 | 26.1 | 24.0 | 30.0 | 4.6 | 5.6 | 3.5 | 4.4 |
| **Wealth decile** | D1 | 10.6 | 21.1 | 14.0 | 17.7 | 4.7 | 4.5 | 3.2 | 3.3 |
|  | D2 | 13.9 | 15.7 | 17.1 | 18.6 | 4.4 | 4.3 | 3.4 | 2.5 |
|  | D3 | 13.2 | 15.9 | 14.7 | 23.0 | 4.7 | 5.7 | 3.6 | 2.7 |
|  | D4 | 11.7 | 14.6 | 16.8 | 17.3 | 4.4 | 5.9 | 2.8 | 2.8 |
|  | D5 | 13.4 | 16.8 | 14.4 | 22.9 | 4.2 | 5.9 | 2.8 | 2.6 |
|  | D6 | 12.4 | 15.1 | 17.1 | 27.3 | 4.4 | 4.8 | 3.8 | 4.1 |
|  | D7 | 13.4 | 18.7 | 18.4 | 68.4 | 4.1 | 4.7 | 2.4 | 3.7 |
|  | D8 | 17.7 | 21.3 | 21.9 | 33.7 | 3.9 | 4.4 | 3.2 | 4.8 |
|  | D9 | 23.2 | 18.2 | 26.2 | 32.2 | 4.2 | 5.0 | 2.8 | 4.0 |
|  | D10 | 32.4 | 22.6 | 31.4 | 31.5 | 3.8 | 4.0 | 3.3 | 3.9 |
| **Province - wealth tertile** | Central- T1 | 17.6 | 30.6 | 35.7 | 36.9 | 10.6 | 14.8 | 11.5 | 7.3 |
|  | Central- T2 | 28.1 | 26.3 | 22.3 | 44.0 | 8.2 | 10.7 | 8.7 |  |
|  | Central- T3 | 25.1 | 45.1 | 41.8 | 69.3 |  | 8.5 | 9.9 | 3.9 |
|  | Copperbelt- T1 | 58.1 | 64.0 | 35.1 | 64.5 |  | 11.4 | 10.7 |  |
|  | Copperbelt- T2 | 20.3 | 25.6 | 32.6 | 36.0 | 10.3 | 19.5 |  | 5.2 |
|  | Copperbelt- T3 | 26.7 | 20.5 | 33.5 | 33.9 | 4.3 | 5.9 | 5.7 | 3.1 |
|  | Eastern- T1 | 18.2 | 19.6 | 15.6 | 23.2 | 6.4 | 5.3 | 4.0 | 4.7 |
|  | Eastern- T2 | 23.5 | 21.8 | 20.2 | 28.8 | 4.8 | 6.8 | 4.5 | 2.5 |
|  | Eastern- T3 | 39.8 | 51.1 | 44.7 | 71.2 | 6.1 | 8.5 |  |  |
|  | Luapula- T1 | 22.2 | 32.0 | 29.7 | 22.6 | 17.8 | 26.0 | 8.4 | 6.7 |
|  | Luapula- T2 | 15.6 | 23.8 | 27.3 | 23.7 | 7.6 | 9.4 | 8.8 | 6.8 |
|  | Luapula- T3 | 51.2 | 46.3 | 43.7 | 54.9 | 13.6 | 8.2 | 4.9 |  |
|  | Lusaka- T1 | 103.4 | 45.4 | 87.1 | 112.3 |  | 13.1 | 10.0 |  |
|  | Lusaka- T2 | 27.2 | 42.9 | 42.0 | 61.9 | 12.9 | 11.6 | 9.0 | 4.3 |
|  | Lusaka- T3 | 20.0 | 18.0 | 23.6 | 29.1 | 2.6 | 6.2 | 5.2 | 2.8 |
|  | Muchinga- T1 | NA | NA | 19.5 | 28.2 | NA | NA | 7.7 | 8.4 |
|  | Muchinga-T2 | NA | NA | 26.1 | 33.6 | NA | NA | 9.5 | 6.7 |
|  | Muchinga-T3 | NA | NA | 47.3 | 78.7 | NA | NA | 6.8 |  |
|  | North Western- T1 | 17.8 | 25.0 | 30.4 | 40.3 | 9.1 | 12.6 | 4.3 | 5.9 |
|  | North Western- T2 | 24.0 | 36.5 | 27.6 | 64.6 | 8.0 | 11.3 | 5.6 |  |
|  | North Western- T3 | 68.3 | 60.9 |  |  |  | 10.3 | 7.6 |  |
|  | Northern- T1 | 13.7 | 19.2 | 21.2 | 39.1 | 9.0 | 10.7 | 10.0 | 7.7 |
|  | Northern-T2 | 17.5 | 25.3 | 24.3 | 57.4 | 7.8 | 13.1 | 4.7 |  |
|  | Northern-T3 | 40.0 | 28.1 | 59.1 | 48.4 |  | 7.6 |  |  |
|  | Southern- T1 | 22.7 | 38.2 | 36.2 | 41.9 | 9.5 | 6.7 | 7.7 | 7.6 |
|  | Southern- T2 | 24.8 | 24.1 | 22.8 | 33.4 | 8.1 | 6.7 | 5.2 | 6.7 |
|  | Southern- T3 | 63.9 | 35.8 |  |  |  | 4.5 | 4.9 |  |
|  | Western- T1 | 17.6 | 28.9 | 48.9 | 31.9 | 11.0 | 10.8 | 9.5 | 9.2 |
|  | Western- T2 | 39.2 | 30.6 | 36.0 | 39.3 | 11.4 |  | 5.7 | 9.1 |
|  | Western- T3 | 26.8 | 104.7 |  |  |  |  | 4.2 | 6.3 |
|  | Green: below 20% threshold indicating good precision | | | |  |  |  |  |  |
|  | Yellow: 20%-40%, indicating fair precision | | | |  |  |  |  |  |
|  | Red: above 40%, indicating poor precision | | | |  |  |  |  |  |
